# Microbes in the built environment

**DOI:** 10.1038/s41598-022-12254-w

**Published:** 2022-06-01

**Authors:** Lena Ciric

**Affiliations:** Healthy Infrastructure Research Group, UCL Department of Civil, Environmental and Geomatic Engineering, Gower Street, London, WC1E 6BT UK

**Keywords:** Water microbiology, Air microbiology, Soil microbiology

## Abstract

The COVID-19 pandemic has encouraged scientists and the general population to think more than ever before about how we interact with microbes in our indoor spaces. Research investigating transmission of SARS-CoV-2 has advanced our knowledge significantly in the last two years. However, indoor and built environment microbiomes are extremely complex polymicrobial systems. We have barely scratched the surface in our understanding of the microbial inhabitants of our indoor and urban spaces. The Microbes in the Built Environment Collection showcases recent research in this important topic around the globe.

In September 2018, I attended a meeting for editorial members at Springer Nature where we were told about the opportunity to guest edit a collection of articles in Scientific Reports. A few weeks later I proposed to be the guest editor for a collection on Microbes and the Built Environment, and we started collecting manuscripts in spring of 2019. Little did we know at the time that the behaviour of microbes in indoor spaces would become such a crucial topic by the end of the year.

The COVID-19 pandemic has brought attention to how microbes circulate around the places we spend our time—whether at home, at work, or in public spaces; indoors and outdoors. There has also been much focus on the removal of microbes in indoor environments, from surfaces and the air, in order to reduce the risk of SARS-CoV-2 transmission^1–3^. The pandemic has resulted in an influx of new research investigating microbial survival and transmission pathways^4–6^. We might therefore think that we have greatly expanded our understanding of the microbes in the built environment beyond what we knew pre-pandemic. However, this assumption would be a mistake. While we know a lot more about SARS-CoV-2 viability and transmission, we have likely neglected the many other microbial inhabitants in the built environment.

Over the past decades, much research has been carried out on the microbial communities present in natural environments such as soil^7^, freshwater systems^8^, marine systems^9^ and the microbiomes of various animals, including humans^10^. As mentioned, however, the microbial communities found in the built environment, such as indoor environments, public spaces, and air and water handling systems, are often neglected. We tend to think that these environments are relatively microbe-free, but this is not the case. Studying these communities, and not just single species of microbes, allows us to better understand the ecological and evolutionary factors that influence them, as well as how they may interact with ourselves and other animals.

We tend to think of microbes as dirty and dangerous, but microbes have been around for billions of years and carry out many useful functions in their various niches. However, we still lack a full understanding of the complex and intricate relationships that various microbial species have between themselves and their environments. This is particularly true in the built environment, where our focus is typically on the removal of microbes through cleaning, due to the possible presence of pathogenic organisms that can change our lives dramatically. SARS-CoV-2 is an example of such an organism, as are the many antibiotic resistant pathogens that have emerged over the past decades^11^. However, there are also species present in our indoor environments which have positive roles, for instance, aiding the development of our immune systems in childhood, and outcompeting pathogens^12^.

We know little about the varied microbial communities present in our built environments—the bacteria, fungi, viruses, algae that we share our spaces with. This Collection is an attempt to get to know them better by curating manuscripts of the state-of-the-art research being carried out to better understand these communities around the world. The Collection includes a variety of microbial communities as well as a broad range of environments in which they are found.

The microbiome present in dust collected from various points within a building can reveal the impact of the environment on microbial community structure. Nygaard et al.^13^ show the differences in the bacterial microbiomes found on floor dust, and on heating ventilation and air conditioning (HVAC) intake and exhaust filter dust, using high-throughput sequencing. They show the HVAC intake filter communities to be rich in environmental outdoor organisms that are associated with the natural environment, such as *Pseudomonas* and *Sphingomonas*. On the other hand, the floor dust and dust from the HVAC exhaust filters were dominated by genera associated with human skin and the respiratory system, such as *Streptococcus* and *Staphylococcus*. Their work shows the influence that occupancy has on the microbiome in indoor spaces.

Hand wash sinks in the healthcare setting were shown to be different in patient rooms and healthcare staff areas. Franco et al.^14^ used bacterial culture methods to look for opportunistic pathogens present in hand wash sinks. They found the drain to be a reservoir for pathogens and high levels of antibiotic resistance among the isolated organisms, and that these organisms persisted despite a rigorous daily cleaning regime. Furthermore, the resistant organisms were more frequently found in patient rather than staff sinks.

Surfaces in various rooms in the homes of older adults were sampled by Sharpe et al.^15^. The samples were then examined using microbiological culture methods. The results showed that the communities present on the surfaces varied according to room and touch frequency. Bedside tables tended to have higher bacterial counts and greater biodiversity when compared to other surfaces, such as the phone, bathroom door, and TV remote. The study also showed that homes where the occupants reported opening windows frequently had different microbial profiles to those who kept windows closed.

Bioaerosols collected from outdoor urban and suburban areas in Japan were shown to have differing microbiomes by Tanaka et al.^16^, according to both location, but also aerosol size. Urban bioaerosols were found to be dominated by human skin-associated bacteria, whereas suburban samples were dominated by soil and plant associated bacteria. There was also a difference in the microbes identified in coarse larger particles and fine smaller particles. The data suggests that local environmental factors, including indoor spaces, could influence outdoor bacterial communities.

And we must not forget the eukaryotes. Lloyd et al.^17^ sampled various freshwater systems in urban Singapore, cultured these for microalgae, and compared the species found in different areas of the city. All samples were found to have microalgae present, and some water bodies have particularly diverse algal communities. The presence of these algae not only contribute to the biodiversity in the high-rise environment of Singapore, but many of the isolated species are also highly effective at carbon-capture, and of commercial significance.

Finally, Chen et al.^18^ show that microbial communities in nasal passages can be influenced by the ventilation strategy within a space. Their study investigated the bacterial communities found in the nares of occupants and the surfaces within spaces using high-throughput sequencing in two groups—hospitalised patients and a non-hospitalised group. The study showed that the nasal and surface communities were more similar in mechanically ventilated spaces. Furthermore, hospitalised patients were more likely to harbour microbes found on their surrounding surfaces highlighting the importance of the patients’ environment.

The “Microbes in the Built Environment” Collection is still open to new submissions. We are interested in work that describes the distribution and viability of microbial communities in built and managed environment contexts, such as buildings, water treatment facilities, public transport and leisure and hospitality venues.
